# Expectation of irrelevant novel stimuli has no consistent effect on recognition memory

**DOI:** 10.1111/sjop.12807

**Published:** 2022-04-07

**Authors:** Richárd Reichardt, Péter Simor, Bertalan Polner

**Affiliations:** ^1^ Department of Cognitive Science Budapest University of Technology and Economics Budapest Hungary; ^2^ Institute of Psychology Eötvös Loránd University Budapest Hungary; ^3^ UR2NF, Neuropsychology and Functional Neuroimaging Research Unit at CRCN – Center for Research in Cognition and Neurosciences and UNI ‐ ULB Neurosciences Institute Université Libre de Bruxelles (ULB) Brussels Belgium; ^4^ Institute of Behavioural Sciences Semmelweis University Budapest Hungary

**Keywords:** Memory, novelty, expectation, recognition

## Abstract

Novelty is defined as the part of an experience that is not yet represented by memory systems. Novelty has been claimed to exert various memory‐enhancing effects. A pioneering study by Wittmann *et al*. (2007) has shown that memory formation may even benefit from the expectation of novelty. We aimed to replicate this assumed memory effect in four behavioral studies. However, our results do not support the idea that anticipated novel stimuli are more memorable than unexpected novelty. In our experiments, we systematically manipulated the novelty predicting cues to ensure that the expectations were correctly formed by the participants, however, the results showed that there was no memory enhancement for expected novel pictures in any of the examined indices, thus we could not replicate the main behavioral finding of Wittmann *et al*. (2007). These results call into question the original effect, and we argue that this fits more into current thinking on memory formation and brain function in general. Our results are more consistent with the view that unexpected stimuli are more likely to be retained by memory systems. Predictive coding theory suggests that unexpected stimuli are prioritized by the nervous system and this may also benefit memory processes. Novel stimuli may be unexpected and thus recognized better in some experimental setups, yet novelty and unexpectedness do not always coincide. We hope that our work can bring more consistency in the literature on novelty, as educational methods in general could also benefit from this clarification.

## INTRODUCTION

From an information‐processing perspective, novelty refers to those elements of an experience that are not represented in the observer's memory systems (Barto, Mirolli & Baldassarre, 2013). Even though this is not the only definition of novelty (Barto *et al*., 2013; Ranganath & Rainer, 2003; Schomaker & Meeter, 2015), it is the so‐called common sense definition, and probably due to its intuitiveness, it tends to guide experimental investigations of novelty in cognitive neuroscience (Schomaker & Meeter, 2018; Schott, 2006; Tulving, Markowitsch, Craik, Habib & Houle, 1996; Wittmann, Bunzeck, Dolan & Düzel, 2007). Since novelty is defined relative to the contents of memory, the cognitive neuroscientific study of novelty is intimately related to memory research.

According to the novelty/encoding hypothesis, the encoding of novel information is different from that of familiar events and leads to superior recognition memory performance (Tulving *et al*., 1996; Tulving & Kroll, 1995). The novelty/encoding hypothesis is built upon the assumption that new information generates novel neural representations with higher excitability compared to already existing ones. Consequently, the associated memory representations are more easily reactivated, leading to higher recognition performance for novel items (Tulving & Kroll, 1995). However, empirical studies produced inconsistent findings in this regard, suggesting that novelty may not always be as relevant for memory encoding as it had been assumed (Dobbins, Kroll, Yonelinas & Liu, 1998; Kormi‐Nouri, Nilsson & Ohta, 2005; Poppenk, Köhler & Moscovitch, 2010).

In sharp contrast, the bulk of animal studies support the idea that novelty has a significant impact on several physiological correlates of memory processes (Lisman & Grace, 2005; Lisman, Grace & Duzel, 2011). Electrophysiological and molecular changes have been shown to occur in the hippocampi of rats placed in novel environments: for example, a study has revealed an increase in the inducibility and the longevity of long‐term potentiation (LTP) in the dentate gyrus (Davis, Jones & Derrick, 2004; Straube, Korz & Frey, 2003), while another experiment has demonstrated a dopamine‐receptor dependent increase in the inducibility of LTP in the CA1 region of the hippocampus (Li, Cullen, Anwyl & Rowan, 2003). These studies used behaviorally relevant stimuli, as the animals were placed in a novel environment, which reliably elicits engagement, or in other words, exploration. More importantly, these experiments were never repeated with the same animals (Li *et al*., 2003; Straube, Korz, Balschun & Frey, 2003), ensuring the novelty factor of the manipulations, which is in strong contrast with studies done with humans that tend to utilize repetitive experimental procedures. Recently, we have suggested that the discrepancy between animal and human data on the memory effects of novelty may stem from the influence of a previously overlooked variable: expectedness (Reichardt, Polner & Simor, 2020).

These animal studies nevertheless inspired a fruitful line of research, which showed that novelty activates the dopaminergic cells of the midbrain (Düzel, Habib, Rotte, Guderian, Tulving & Heinze, 2003; Köhler, Danckert, Gati & Menon, 2005; Kumaran & Maguire, 2006, 2007; Schott, Sellner, Lauer *et al*., 2004). Such findings lead to the idea that novelty is itself rewarding, because it represents potentially useful information which increases the chance of survival (Düzel, Bunzeck, Guitart‐Masip & Düzel, 2010; Lisman, Grace & Duzel, 2011). Yet if we subscribe to the idea that novelty is valuable because of its usefulness we find a contradiction in the result that even irrelevant novelty influences the brain and cognition in the same way as rewards do. A study by Wittmann *et al*. (2007) suggests that even the anticipation of irrelevant novel stimuli enhances memory formation. In their study, Wittmann and colleagues used fMRI to assess brain activation patterns in response to expected and unexpected novel pictures. The main finding was that as expected, novel pictures elicited increased activity in the midbrain. Presumably, dopaminergic neurons are in part responsible for this activation and through a connection to the hippocampus they could affect memory encoding. To confirm this hypothesis, the authors conducted a small laboratory experiment (*N* = 12). The participants repeated the same procedure as the fMRI group, but after a 24 h delay they returned to the laboratory for a surprise recognition memory test. During the memory test, the participants also gave remember/know responses in addition to the recognition responses. The results showed that the ratio of remember and know responses were different for expected and unexpected novel pictures: relatively more remember responses were given for expected novel pictures. This result was taken to support the idea that novelty is processed akin to rewards by the brain and expectation of novelty results in dopamine release in the hippocampus and improved memory performance just like the expectation of reward (Adcock, Thangavel, Whitfield‐Gabrieli, Knutson & Gabrieli, 2006). The stimuli employed in this study are uninteresting in themselves (black and white pictures of landscapes) and irrelevant for future goals, because the participants only have to judge if the stimulus presented is one of a few familiars or a novel one, but are not instructed to memorize them. The result that the anticipation of stimuli that has no future relevance still enhances memory formation on the one hand supports a simple interpretation of the idea that novelty is inherently rewarding, but seems to be at odds with the predictive coding framework applied to memory systems: the learning of unexpected events should be prioritized by the brain (Clark, 2013; Friston, 2010).

Since the memory effects of novelty are somewhat contentious in human neuroscience (Barto *et al*., 2013; Reichardt *et al*., 2020; Schomaker & Meeter, 2015) we wanted to assess if the behavioral result reported by Wittmann and colleagues can be replicated on a larger sample. Novel ideas on the memory effects of novelty suggest that it is the quality of unexpectedness that is critical for memory formation (Frank & Kafkas, 2021; Quent, Henson & Greve, 2021; Reichardt *et al*., 2020). We hypothesized that in the paradigm introduced by Wittmann and colleagues, unexpected novelty should be more memorable than expected novelty, because the degree of unexpectedness is generally larger for unexpected novelty. Here, we present the findings of four studies we conducted using the paradigm of Wittmann and colleagues, with minor modifications to ensure that the participants behaved as intended.

## STUDY 1

We first calculated the effect size for the memory effect reported in the original study. The mean difference of corrected remember minus know rates for expected novel pictures was 8.9 (±5) and 0.9 (±4) for unexpected novel pictures. We calculated the effect size for the *t*‐test used to compare these means (Cohen's *d* = 1.77). Then we calculated the statistical power (0.99) for this analysis with the number of participants in the Wittmann study (*n* = 12) with alpha set to 0.05. Therefore, we concluded that even a sample of 10 participants should be enough to replicate this effect with high statistical power. During a pilot study with 10 participants, however, we found no statistically significant difference between any indices of recognition memory. We speculated that the original study may have overestimated the true effect size, and we decided to aim for ~30 participants in our studies, which, with alpha set to 0.05, would yield circa 90% power to detect an effect that is smaller than what was reported in the original study (*d* = 0.61).

### Methods

We based our studies on a paradigm by Wittmann *et al*. (2007). The task consists of three phases, two of which are completed on the same day, and one after a 24 ± 2 h delay. The first phase is the familiarization phase, where the participants are repeatedly shown images of black and white landscapes. The second phase starts immediately after the familiarization. It consists of a cued stimulus presentation sequence, where a cue predicts familiar and another predicts novel pictures. The participants have to indicate with a fast button press if the currently seen picture is familiar or new. This phase is called the study phase, because it is an incidental learning phase: unbeknownst to the participants, their memory for the novel pictures seen during this phase will be tested on the next day. The participants return to complete the test phase after 24 h.

#### Participants

Participants were undergraduate students of the Budapest University of Technology and Economics reporting no prior or current psychiatric, neurological or chronic somatic disorders (*N* = 39). Participants received partial credit points for completing the tasks. The data of three participants were excluded due to extremely low overall recognition rates (below 5%). This way the data of 36 participants (25 females, mean age ± standard deviation: 21.8 ± 6 years) was analyzed. A sensitivity analysis indicated that the study could detect a minimum effect of *d* = 0.56 with 90% power and alpha set to 0.05. The study was approved by the Hungarian Ethical Review Committee for Research in Psychology, and participants provided written informed consent before completing the tasks.

#### Procedure

**Fig. 1:**
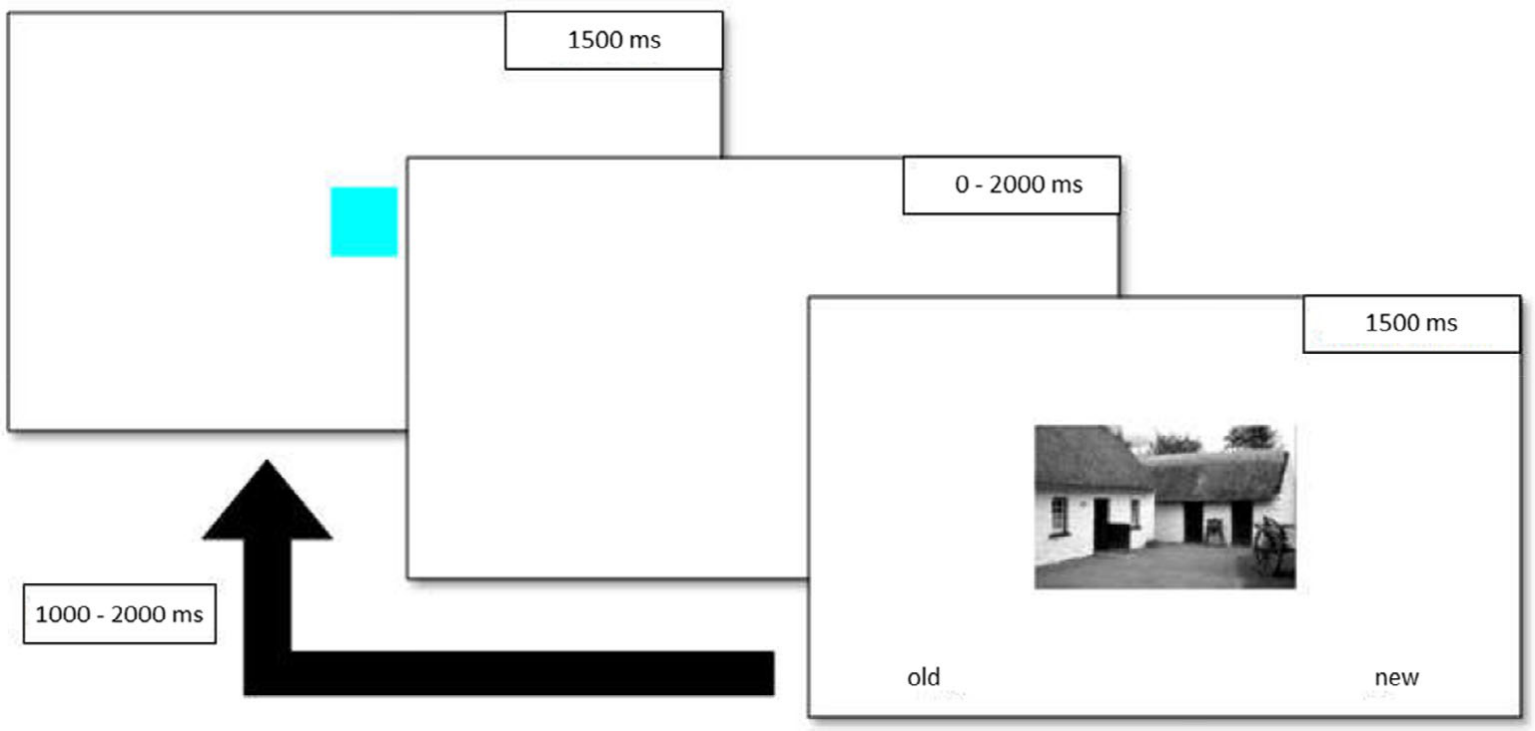
The structure of trials during the study phase. A cue indicating the category of the next picture is followed by variable delay. The participants have to indicate the category of the picture with a button press, as fast as they can. They have a maximum of 1,500 ms to respond and the pictures are visible for this duration. [Colour figure can be viewed at wileyonlinelibrary.com]

The task was programmed in the OpenSesame environment (Mathot, Schreij & Theeuwes, 2012). The tasks were completed in two sessions, separated by 24 ± 2 h. The design consisted of cued stimulus presentation on the first day followed by a recognition memory test on the second day. Stimuli were black and white landscapes that were made available by the authors of the original study. We selected 186 pictures from this stimulus pool, which were randomly assigned to different stimulus categories (familiar or novel, detailed below) for each participant. At the beginning of the first session, participants were familiarized with six pictures. These were presented 10 times each for a duration of 1,500 ms in a randomized order. Participants were instructed to memorize these pictures. This familiarization phase was followed by the study phase with 240 trials. A trial consisted of the presentation of a cue (1,500 ms), followed by a delay with blank screen (0–2,000 ms), the presentation of a picture (1,500 ms) and the intertrial interval (1,000–2,000 ms – see Fig. 1 for a diagram of the study phase). There were two cues, a yellow and a cyan square, presented at the center of the screen. One of these predicted the familiar and the other the new picture category with 75% accuracy. The cues were randomly assigned to a category at the start of the experiment and the associated category and their accuracy were made clear to the participants through the instructions (e.g., “the yellow square will mostly be followed by a familiar picture”). The cue was followed by the presentation of one of the familiarized pictures or a novel picture. This 2 × 2 (novelty × expectation) design produced four categories of trials, which were: expected familiar (90 trials), unexpected familiar (30 trials), expected new (90 trials), and unexpected new (30 trials) pictures. The task of the participants was to indicate as quickly as possible (by a button press) whether the picture was familiar or new. A maximum duration of 1,500 ms was provided for a response. During the pilot testing of the experiment, we discovered that participants frequently ignored the symbolic cues, as they could discriminate familiar vs. novel pictures easily without paying attention to the cue. In order to increase the relevance of the cues, we fixed the first 24 trials to be congruent, that is, a given cue was always followed by the corresponding stimulus category to ensure that the participants learn the contingencies and pay attention to the cues. We also decreased the number of trials from 320 to 240 to reduce the length of the experiment and to increase the recognition rates, which were extremely low (below 0.1) in the pilot studies.

**Fig. 2:**
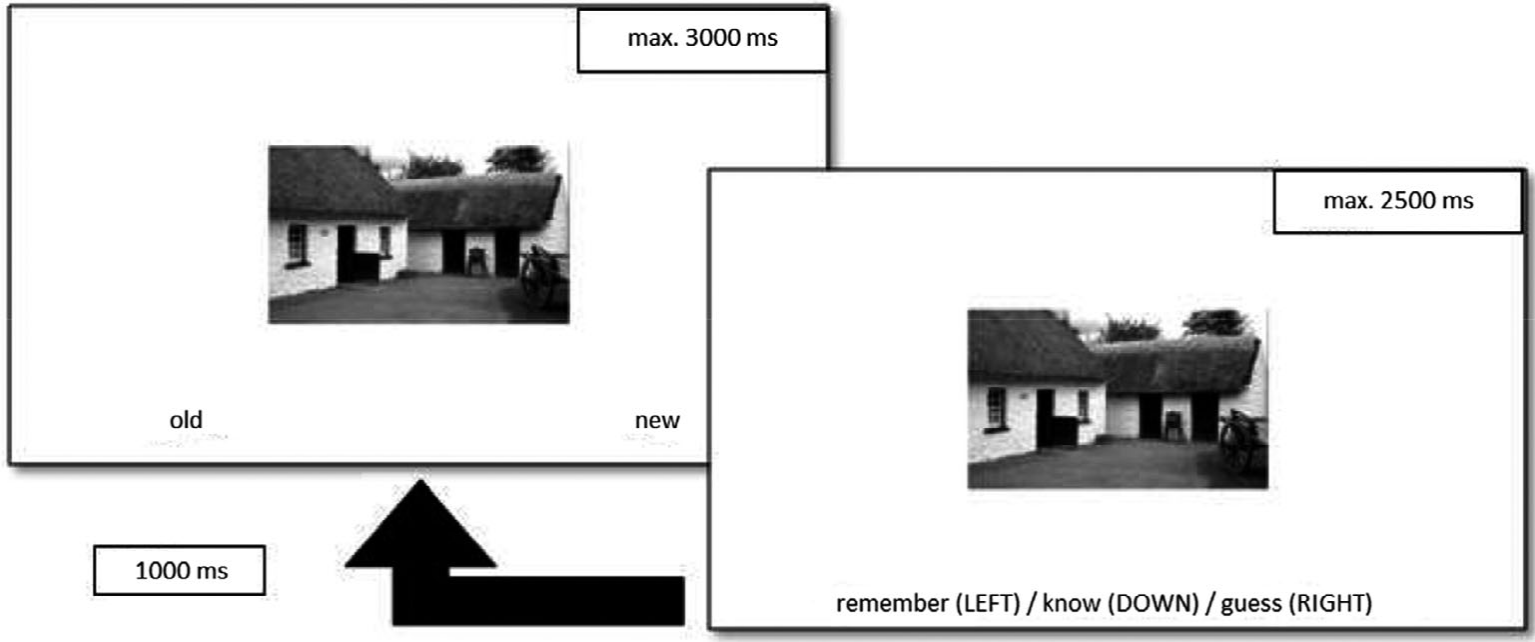
The structure of trials during the test phase. The participants have to indicate with a button press if they think they saw the picture during the study phase (“old”) or not (“new”). In Study 1 and 2 the participants had to give a remember/know response after every “old” response, while in Study 3 and 4 they gave a confidence rating.

On the second occasion (24 ± 2 h after the first session), participants completed a recognition memory test, during which they were shown the previously presented novel pictures interspersed with 60 distractor pictures. The pictures were presented one at a time, and the participants had to indicate with a button press whether they did or did not see the picture in the study phase (“old” vs. “new” responses). The participants had 3,000 ms to respond. If they gave an “old” response, they had to indicate the quality of their memory with a “remember/know” judgement within 2,500 ms. There was also a “guess” option to decrease the noise from accidental button presses and unsure responses. The intertrial interval was set to 1,000 ms in this phase (see Fig. 2 for a visual description of the test phase). Importantly, participants were not instructed to memorize the pictures presented in the study phase, thus, the recognition memory test on the second day gives a measure of incidental learning. This ensures that the experimental setup measures the impact of natural sources of salience (supposedly novelty) without adding any further source of salience inherent to the task design.

Overall, Study 1 can be viewed as a direct replication of the behavioral part (“Separate memory assessment”) of Wittmann *et al*., 2007. We further reduced the number of trials to increase performance during the test (Wittmann *et al*., 2007: 120 expected familiar, 40 unexpected familiar, 120 expected novel, 40 unexpected novel trials during the study phase; 120 expected novel, 40 unexpected novel and 80 distractor trials during the test phase; Study 1 of the present paper: 90 expected familiar, 30 unexpected familiar, 90 expected novel, 30 unexpected novel trials during the study phase; 90 expected novel, 30 unexpected novel and 60 distractor trials during the test phase). We also reduced the delay between the cue and the stimulus (from 0–4,500 ms to 0–2,000 ms) and the intertrial interval (from 1,500–4,500 to 1,000–2,000 ms) to save time. We actively mislead the participants about the goals of the second session so that they did not anticipate memory testing. In our view, this was crucial, since if participants anticipate memory testing, novelty and task relevance coincide. We structured the presentation during the study phase: we fixed the first 10% of study trials (24) to be either expected familiars of expected novels. This was done in order to facilitate learning the link between the cue and the category it predicts. In our view, this change ensures that expectations form correctly during the task. Finally, our participants gained course credits for their participation, while Wittmann and colleagues used monetary compensation.

#### Statistical analyses

All analyses were done using R (R Core Team, 2020) in R Studio (RStudio Team, 2015) and data visualization was performed with the ggplot2 package (Wickham, 2016). Power and sensitivity analyses were performed with GPower 3.1 (Erdfelder, Faul, Buchner & Lang, 2009).

For the study phase, we calculated the mean accuracy (proportion of correct old or new responses) as well as the average reaction times for the four different trial categories (expected familiar, unexpected familiar, expected novel, unexpected novel) in the study phase and used analysis of variance (ANOVA) to assess the effect of trial type (old vs. new) and expectation (expected vs. unexpected) on these variables. Accuracy shows if the participants were able to memorize the familiar pictures and correctly discriminate these from novel stimuli, while reaction times can be used to assess the effect of the cues.

For the test phase, we calculated the proportion of remember/know responses for expected and unexpected novel pictures for every participant. First, we summed up each response type for expected and unexpected novel pictures and divided the result by the number of trials in each category to get uncorrected remember and know rates. Then, we calculated false alarm rates for remember and know responses by counting these responses for distractor pictures. Finally, we derived the corrected remember and know rates for both expected and unexpected novel pictures by subtracting the appropriate false alarm rate from the uncorrected rate. We used an ANOVA to reveal any differences due to response type and expectation. We also computed the Bayes Factor for the critical comparison, that is, corrected remember rates for expected vs. unexpected novel pictures, with the use of the BayesFactor R package (Morey, Rouder, Jamil & Morey, 2015). We used the default Cauchy prior width of *r* = 0.707. We also calculated the Recollection Estimate (RE) and Familiarity Estimate (FE) as per the original study, based on previous recommendations (Yonelinas *et al*., 1996). The RE is basically the same as the corrected remember rate (remember rate minus remember false alarm rate). To get the FE, we first calculated the rate of familiarity by subtracting the RE from the overall hit rate and dividing the result by one minus RE. We then subtracted the *z* score for the familiarity false alarm rate from the *z* score of the familiarity rate. Finally, both the RE and FE were *z* transformed and these values were used in an ANOVA with response type (RE and FE) and expectation (expected and unexpected) as within subjects factors. We also calculated overall recognition rates separately for expected and unexpected items. We used a paired *t*‐test to compare the uncorrected recognition rates for expected and unexpected pictures, and calculated the corresponding Bayes Factor. These analyses were done to reveal any differences between the memory performance for the expected versus the unexpected novel picture category.

### Results

**Fig. 3:**
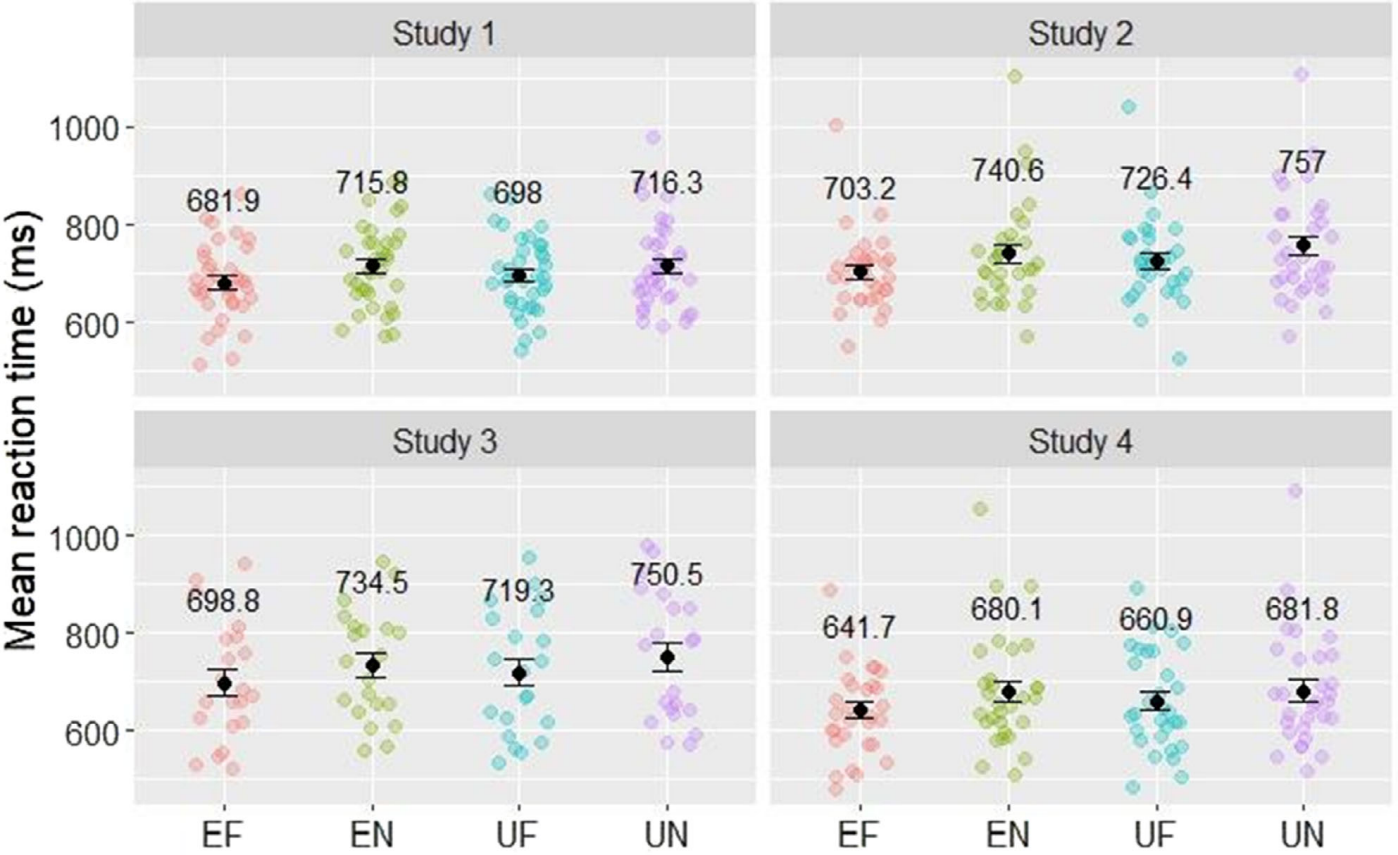
Reaction times during the Study phase for expected familiar (EF), unexpected familiar (UF), expected novel (EN), and unexpected novel (UN) pictures in Study 1–4. Slower responses to unexpected pictures indicate attention to the cues. [Colour figure can be viewed at wileyonlinelibrary.com]

Accuracy during the study phase was over 95% for all categories (see Table 1) and an ANOVA revealed no main effects for or interaction between expectedness and picture type (STIMULUS CATEGORY: *F*[1,35] = 1.39, *p* > 0.1; EXPECTATION: *F*[1,35] = 2.06, *p* > 0.1; EXPECTATION × STIMULUS CATEGORY interaction: *F*[1,35] = 0.11, *p* > 0.5), suggesting that participants were able to memorize the familiar pictures and could successfully distinguish them from novel pictures. We also analyzed the reaction times for the four stimulus categories during the study phase (see Fig. 3 for the mean reaction times in all studies). A two‐way ANOVA showed a significant effect of STIMULUS CATEGORY (*F*[1,35] = 7.60, *p* = 0.01, η^2^ *G* = 0.025) but not of EXPECTATION (*F*[1,35] = 2.96, *p* = 0.09, = 0.003) indicating quicker responses for familiar items. Moreover, the interaction between stimulus category and expectation was also significant (*F*[1,35] = 4.81, *p* < 0.05, η^2^ *G* = 0.002). Parsing out this interaction revealed that the reaction times for expected familiar pictures were significantly lower compared to all other trial categories (unexpected familiar: *p* < 0.05, expected new: *p* < 0.01, unexpected new: *p* = 0.01), while the response times between unexpected familiar and any new trial types or expected and unexpected new trials did not differ (*p* > 0.1 for all of these pairwise comparisons).

**Table 1:** Means and standard deviations for the variables of interest in the study phase in the behavioral study of Wittmann et al. (2007) and Study 1–4

	Study phase							
	Accuracy (%)				Reaction time (ms)			
	EF	UF	EN	UN	EF	UF	EN	UN
Wittmann *et al*. (2007)	93.8 ± 3.9	93.4 ± 3.5	95.1 ± 3.7	94.1 ± 3.6	602 ± 28	642 ± 40	687 ± 31	713 ± 34
Study 1	97.6 ± 3.5	96.9 ± 4.7	98.3 ± 1.7	98.0 ± 3.3	681.9 ± 77.4	698.0 ± 77.4	715.8 ± 84.7	716.3 ± 89.4
Study 2	98.1 ± 1.9	97.8 ± 3.2	98.1 ± 2.1	97.0 ± 4.7	703.2 ± 80.1	726.4 ± 88.9	740.6 ± 105.8	757.0 ± 111.2
Study 3	96.1 ± 4.2	94.8 ± 4.5	95.1 ± 5.2	95.6 ± 4.4	698.8 ± 122.6	719.3 ± 127.8	734.5 ± 113.0	750.5 ± 133.1
Study 4	96.7 ± 3.3	96.9 ± 4.8	97.3 ± 2.7	97.0 ± 3.4	641.7 ± 92.2	661.0 ± 100.6	680.1 ± 116.4	681.8 ± 118.1

*Note*: EF = expected familiar; EN = expected novel; UF = unexpected familiar; UN = unexpected novel.

**Fig. 4:**
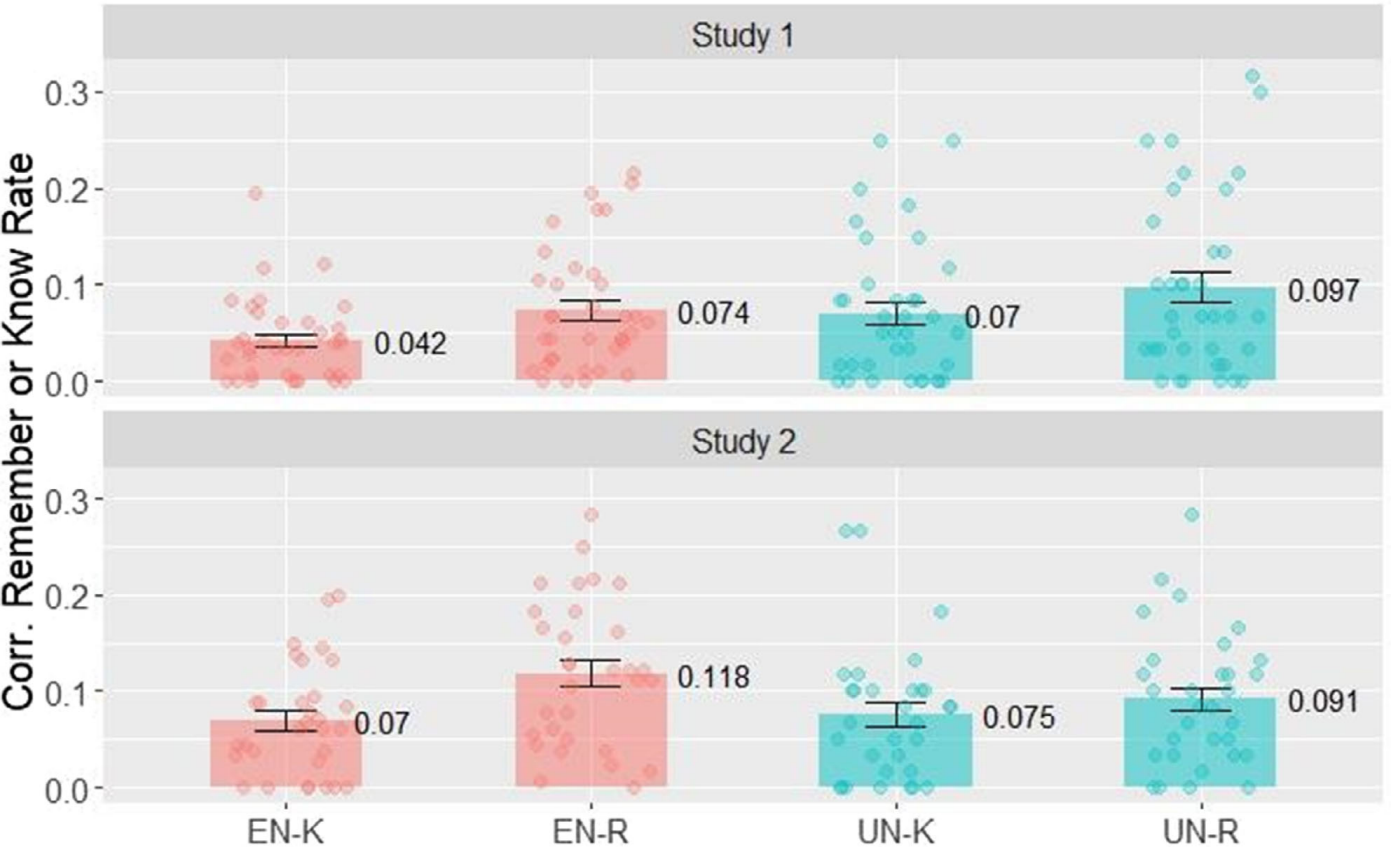
Corrected Know and Remember rates for expected (EN‐K and EN‐R) and unexpected (UN‐K and UN‐R) novel pictures in the Test Phase in study 1–2. Previous reports indicated that the ratio of remember and know responses are higher for expected novel pictures, however, this effect does not appear in Study 1 or 2. [Colour figure can be viewed at wileyonlinelibrary.com]

**Fig. 5:**
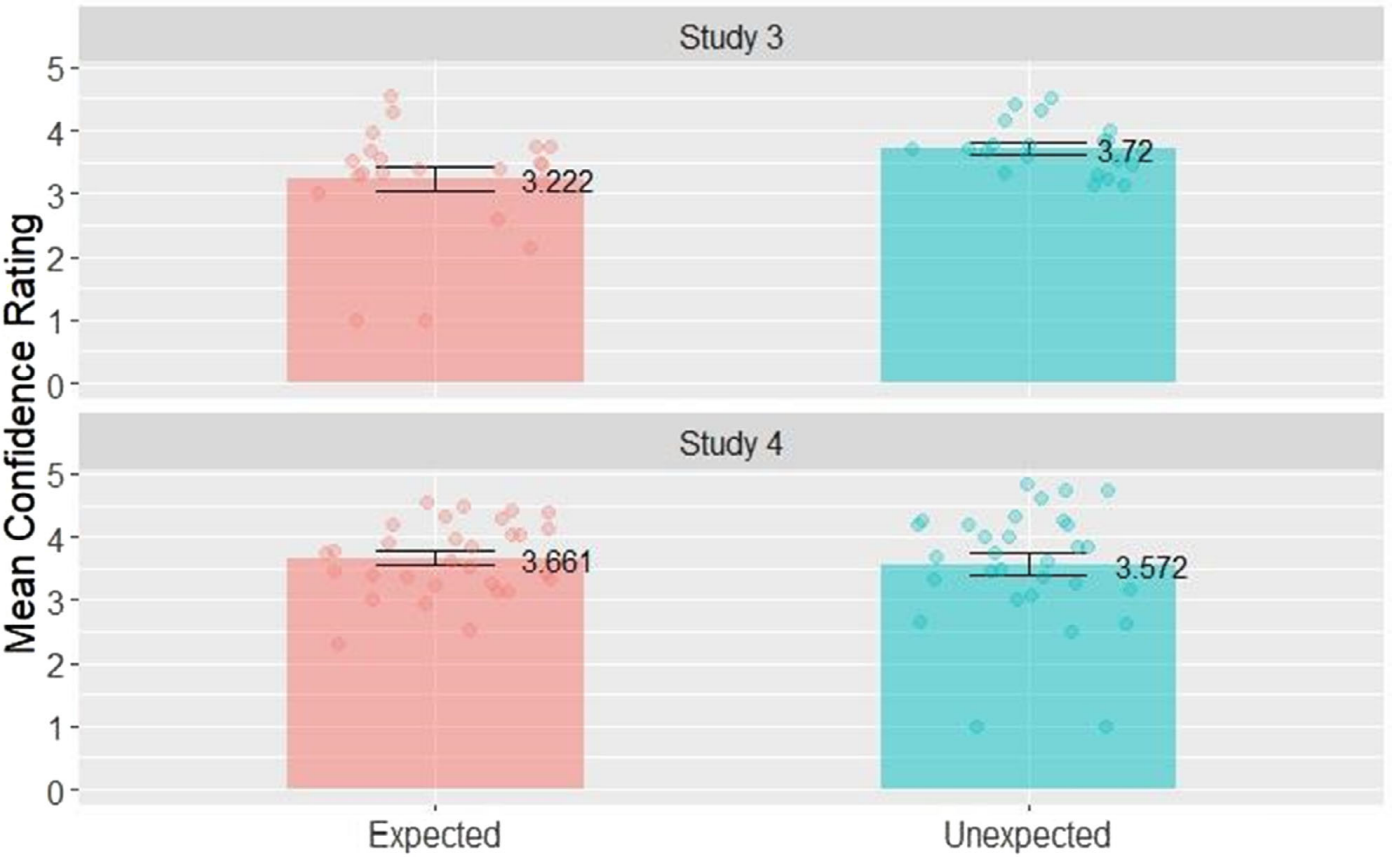
Uncorrected recognition rates for expected and unexpected novel pictures in the Test Phase in Study 1–4. The overall recognition rates do not differ significantly in Study 2, 3, and 4, yet, in Study 1 there is a marked difference in favor of unexpected novel pictures, a result that is at odds with the original results, but more in line with current ideas on memory formation. [Colour figure can be viewed at wileyonlinelibrary.com]

Corrected remember and know rates in Study 1 are shown in Fig. 4. A two‐way ANOVA with factors RESPONSE TYPE (levels: remember, know) and EXPECTATION (levels: expected, unexpected) showed no significant difference in the proportion of corrected remember and know responses (RESPONSE TYPE: *F*[1,35] = 0.80, *p* > 0.1, η^2^ *G* = 0.009), while EXPECTATION had a significant main effect (EXPECTATION: *F*[1,35] = 8.40, *p* < 0.01, η^2^ *G* = 0.012): remember and know rates were higher for unexpected, compared to expected novel pictures. The interaction between these factors was not significant (RESPONSE TYPE × EXPECTATION interaction: *F*[1,35] = 0.32, *p* > 0.5, η^2^ *G* = 0.001). The Bayes factor for the *t*‐test comparing the remember rates for expected and unexpected novel pictures was 0.409, which can be interpreted as anecdotal evidence for the null hypothesis (Andraszewicz *et al*., 2015). The ANOVA on the RE and FE values also showed a main effect of EXPECTATION (*F*[1,35] = 10.84, *p* < 0.01, η^2^ *G* = 0.018), but no main effect of RESPONSE TYPE (*F*[1,35] < 0.01, *p* = 1.0, η^2^ *G* < 0.001) and no significant interaction between these factors (*F*[1,35] = 0.10, *p* = 0.75, η^2^ *G* < 0.001). We also compared the uncorrected hit rates for expected and unexpected novel pictures (Fig. 5) with a paired *t*‐test (false alarm rates are the same for both categories this way, thus corrected and uncorrected rates yield the same result). A Shapiro–Wilk test did not indicate violation of normality (*p* > 0.05). The paired *t*‐test indicated higher overall recognition for unexpected compared to expected novel pictures (*M* = 0.405, *SD* = 0.132 vs. *M* = 0.357, *SD* = 0.150; *t*(35) = 2.85, *p* = 0.007, *d* = 0.33, CI [0.09, 0.57]). The Bayes factor of the *t*‐test comparing uncorrected recognition rates for expected and unexpected novel pictures was 5.511. Thus, it serves as moderate evidence that memory performance is better for unexpected pictures.

### Discussion

Accuracy during the study phase was high, showing that the participants could reliably distinguish between familiarized and novel pictures. Reaction times were expected to be significantly lower for expected pictures, however, this only appeared as a tendency. There was no significant difference between the remember and know rates per expectation category, yet response rates were higher for unexpected stimuli. We observed the same effects using the recall and familiarity estimates. Thus, the original results are not replicated in this study. The uncorrected recognition rates were significantly higher for unexpected pictures however, which supports the hypothesis derived from the predictive coding framework.

## STUDY 2

As the cues are central to this paradigm, we set out to test how minor changes to the cueing strategy influence the results of the memory test. In study 2, we used verbal cues: texts clearly stating the picture category (‘old/new’) to expect. We hypothesized that a well‐known association between the cue and the stimulus categories would facilitate the effect of cueing.

### Participants

Forty‐one university students with no prior or current history of psychiatric, neurological or chronic somatic disorders participated in the second experiment. The participants were recruited from the Budapest University of Technology and Economics and Eötvös Lóránd University and received partial credit points. Seven subjects had to be excluded by our exclusion criteria: accuracy below 0.8 during the study phase (5), false alarm rate over 0.5 during the test phase (3) and overall uncorrected recognition rate below 0.05. This left 34 participants (25 females, mean age: 20.9 ± 1.5). A sensitivity analysis indicated that the study could detect a minimum effect of *d* = 0.57 with 90% power and alpha set to 0.05. The study was approved by the Hungarian Ethical Review Committee for Research in Psychology, and written informed consents were obtained.

#### Procedure

The general design, the amount of trials and the temporal characteristics of the experiment were the same as in Study 1. However, in Study 2 we applied verbal cues, which directly indicated the category of the next picture (“old” vs. “new”). We implemented this change because we failed to replicate the pattern in reaction times during the study phase reported by Wittmann *et al*. (2007) in Study 1. The association between verbal cues and the picture categories is implicitly known in this case. Even though the cues were self‐describing this way, we still used the trial structuring procedure described for Study 1. During the recognition memory test, we collected ‘remember/know’ responses.

#### Statistical analyses

All procedures were the same as for Study 1.

### Results

The overall accuracy of the participants was also over 95% in this experiment (see Table 1). This again indicates that participants were able to memorize the familiar pictures and to distinguish them from novel pictures. Accuracy was not significantly influenced by stimulus category or expectation, as an ANOVA showed using the same factors as in Study 1 (EXPECTATION: *F*[1,30] = 1.45, *p* > 0.1; STIMULUS CATEGORY: *F*[1,30] = 0.69, *p* > 0.1; EXPECTATION × STIMULUS CATEGORY interaction: *F*[1,30] = 0.74, *p* > 0.1). The mean values of reaction time for the different categories showed a similar pattern to that of Study 1 (see Fig. 3 for the mean reaction times in all studies). A two‐way ANOVA with factors STIMULUS CATEGORY and EXPECTATION yielded a significant main effect for STIMULUS CATEGORY (*F*[1,30] = 11.8, *p* = 0.001, η^2^ *G* = 0.031), indicating faster responses to familiar pictures, and EXPECTATION (*F*[1,30] = 16.7, *p* < 0.001, η^2^ *G* = 0.011), showing faster responses to expected pictures. The STIMULUS CATEGOR × EXPECTATION interaction had no significant effect (*F*[1,30] = 0.57, *p* > 0.1, η^2^ *G* < 0.001).

Corrected remember and know rates are visible in Fig. 4. For the test phase, a two‐way ANOVA with factors RESPONSE TYPE (levels: remember, know) and EXPECTATION (levels: expected, unexpected) showed no significant difference in the proportion of corrected remember and know responses (RESPONSE TYPE: *F*[1,30] = 3.96, *p* > 0.05, η^2^ *G* < 0.001) and neither did expectation (EXPECTATION: *F*[1,30] = 1.45, *p* < 0.1, η^2^ *G* = 0.006) or the interaction of these factors (RESPONSE TYPE × EXPECTATION interaction: *F*[1,30] = 2.62, *p* > 0.1, η^2^ *G* < 0.008). The ANOVA using the RE and FE values did not show main effects (EXPECTATION: *F*[1,30] = 1.70, *p* = 0.20, η^2^ *G* = 0.008; RESPONSE TYPE: *F*[1,30] < 0.01, *p* = 1.0, η^2^ *G* < 0.001) or an interaction effect (*F*[1,30] = 3.14, *p* = 0.09, η^2^ *G* < 0.009). The Bayes factor of the *t*‐test comparing the remember rates for expected and unexpected novel pictures was 0.858, showing anecdotal evidence for the null hypothesis.

The uncorrected hit rates for Study 2 are presented in Fig. 5. We compared the uncorrected hit rates for expected and unexpected novel pictures with a paired *t*‐test (Shapiro–Wilk test of normality *p* > 0.5). The paired *t*‐test indicated no difference between overall uncorrected recognition rates for unexpected and expected novel pictures (*M* = 0.359, *SD* = 0.130 vs. *M* = 0.376, *SD* = 0.117; *t*(30) = 0.43, *p* > 0.1, *d* = 0.13, CI [−0.48, 0.20]). The Bayes factor of the *t*‐test comparing uncorrected recognition rates for expected and unexpected novel pictures was 0.258, showing moderate support for the null hypothesis.

### Discussion

High accuracy during the study phase shows that the required distinction was relatively easy for the participants. The analysis of reaction times showed that the responses to expected and familiar pictures were faster in this study. The main result of the original study was not replicated by this experiment either and no significant difference between the recognition rates for the picture categories was observed. Taken together, these results do not support any hypotheses we tested.

## STUDY 3

In study 3, we used symbolic cues again (cyan and yellow squares); however, we did not fix the first 10% of the trials. We will refer to this trial structure as “unstructured” throughout the text. We also changed the remember/know response to a simple confidence response in this study and in Study 4. This decision was made because a confidence response is far more intuitive than remember/know judgements. Since “remember” responses usually correspond to higher confidence scores, as compared to “know” responses (Yonelinas, Aly, Wang & Coen, 2010), we presumed that the original results would be conceptually replicated in these studies if the mean of the confidence ratings for correct recognitions was significantly higher for expected than for unexpected novel pictures.

### Participants

Twenty‐eight university students free from prior or current history of psychiatric, neurological or chronic somatic disorders participated in the second experiment. The participants were recruited from Eötvös Lóránd University and collected partial course credits. Exclusion of seven subjects was deemed necessary: accuracy below 0.8 during the study phase (3), false alarm rate over 0.5 during the test phase (4) and overall uncorrected recognition rate below 0.05. This left 21 participants (10 females, age: 21.2 ± 2.1). A sensitivity analysis indicated that the study could detect a minimum effect of *d* = 0.74 with 90% power and alpha set to 0.05. The study was approved by the Hungarian Ethical Review Committee for Research in Psychology, and written informed consents were obtained from the participants.

### Procedure

The general design, the amount of trials and the temporal characteristics of the experiment were the same as in Study 1. In Study 3, however, we removed the structuring of trials and used the original symbolic cues (cyan and yellow squares). By removing trial structuring we wanted to more closely imitate the methods described in Wittmann *et al*. (2007). We changed the recognition memory test, so that we did not collect “remember/know” responses in this study, but a confidence rating for recognized items (“How sure are you in your response?” “1: Not at all”; 2: Unlabeled; “3: It may be right”; 4: Unlabeled; “5: Totally”). This change was implemented to ease the instruction of the participants, yet still collect a response related to the metacognitive assessment of memory.

### Statistical analyses

All procedures were the same as for Study 1, apart from analyses relating to remember and know rates as these responses were replaced by confidence ratings. We calculated the mean of the confidence responses for correct recognitions across expected and unexpected picture categories by participant and compared these with a paired *t*‐test (including the Bayes Factor). We also used a Shapiro–Wilk test on the differences of the mean confidence ratings for expected and unexpected pictures, and finally compared the confidence ratings with a Wilcoxon signed rank test. We also used receiver operating characteristics (ROC) curves to compare expected and unexpected picture recognition performance. We first calculated the hit rate and false alarm rate for expected and unexpected pictures of every confidence level for each participant. We then fitted ROC curves for the expected and unexpected categories separately, and compared the area under the curve (AUC) values with DeLong's test using the pROC package for R (Robin, Turck, Hainard, *et al*., 2011).

### Results

Again, in the study phase, the overall accuracy of the participants was over 95% (see Table 1), indicating that participants successfully made the distinction between familiar and novel pictures in most of the trials. A two‐way ANOVA showed no significant main effect or interaction for accuracy (STIMULUS CATEGORY: *F*[1,20] = 0.01, *p* > 0.5; EXPECTATION: *F*[1,20] = 0.37, *p* > 0.5; STIMULUS CATEGORY × EXPECTATION interaction: *F*[1,20] = 1.92, *p* > 0.1). The two‐way ANOVA on reaction times yielded a significant main effect for STIMULUS CATEGORY (*F*[1,20] = 13.36, *p* = 0.001, η^2^ *G* = 0.019), showing faster responses to familiar pictures, while there was no main effect for EXPECTATION (*F*[1,20] = 3.64, *p* = 0.07, η^2^ *G* = 0.006) and also no significant effect for the STIMULUS CATEGORY × EXPECTATION interaction (*F*[1,20] = 0.74, *p* > 0.5, η^2^ *G* < 0.001). The mean values of reaction time for the different categories showed a similar pattern to that of the other experiments (see Fig. 3 for the mean reaction times in all studies).

**Fig. 6:**
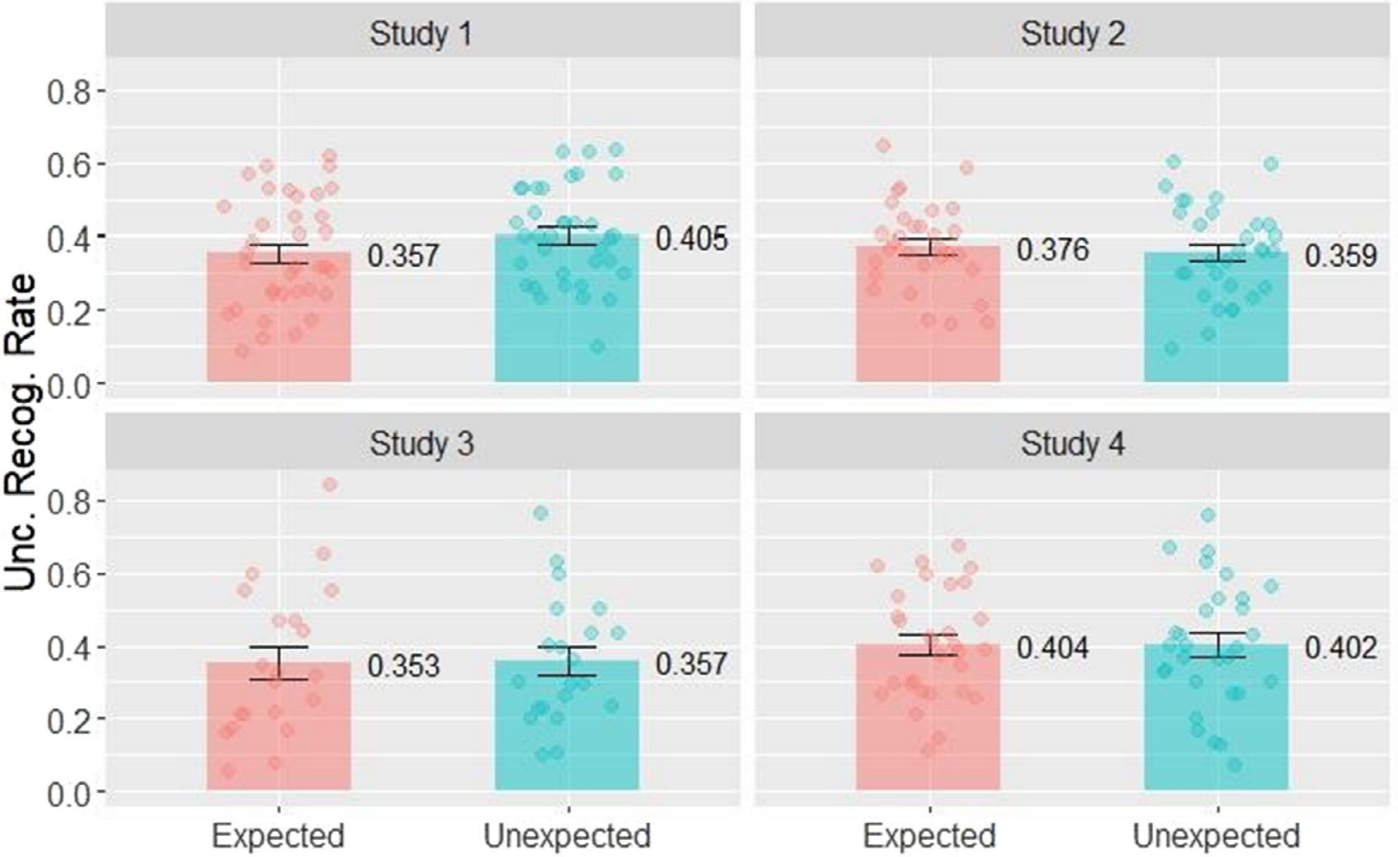
Mean confidence ratings for expected and unexpected novel pictures in the Test Phase in Study 3–4. Confidence ratings are more intuitive and the previously reported greater proportion of remember responses to expected pictures could translate to higher mean confidence rate. This hypothesis is contradicted by our data. [Colour figure can be viewed at wileyonlinelibrary.com]

Regarding the test phase, we compared the means of the confidence ratings for correctly recognized expected and unexpected pictures with a paired *t*‐test and did the same for uncorrected overall recognition rates (see Fig. 5 for uncorrected recognition rates and Fig. 6 for confidence ratings). The confidence ratings were significantly higher for unexpected pictures than for expected stimuli (*M* = 3.72, *SD* = 0.40 vs. *M* = 3.22, *SD* = 0.90; *t*(20) = 2.24, *p* = 0.028, *d* = 0.71, CI [0.03, 1.39]). However, the Shapiro–Wilk test yielded a significant result (*p* < 0.001), thus we compared confidence ratings with the Wilcoxon signed rank test, which showed no significant difference between the categories (*p* = 0.06; Cliff's Delta = 0.38, CI [0.04–0.63]). The Bayes factor of the *t*‐test comparing the mean confidence ratings for expected and unexpected novel pictures was 2.173 (anecdotal evidence that the mean confidence rating for unexpected novel pictures is higher than that of the expected novels). The AUC for the ROC curve fitted to the responses to the expected picture category was 0.61 (95% CI: 0.44–0.78) and for the unexpected pictures was 0.65 (95% CI: 0.51–0.78), meaning that performance was over chance level (0.5), however, there was no significant difference between the AUC in the two conditions (*D* = −0.36, *p* = 0.72). There was no significant difference between the corrected hit rates for unexpected and expected novel pictures (*M* = 0.357, *SD* = 0.174 vs. *M* = 0.353, *SD* = 0.206; *t*(20) = 0.24, *p* > 0.5, *d* = 0.02, CI [−0.13, 0.16]). The Bayes factor of the *t*‐test comparing uncorrected recognition rates for expected and unexpected novel pictures was 0.234, showing moderate support for the null hypothesis.

### Discussion

The results again seem to support the idea that the task during the study phase was relatively easy and the participants paid attention to the cues. The confidence ratings were not higher for expected novel pictures thus the original effect does not appear in this study either. The confidence ratings were nominally higher for unexpected novel pictures; however, the difference was not significant. The overall uncorrected recognition rates were also not significantly different in this study, meaning that neither the original result nor the result of our first study was replicated.

## STUDY 4

We once again used verbal cues (“old/new”) in study 4, but with an unstructured study phase, so that every combination of cue type and trial structuring is tested.

### Participants

Thirty‐five university students free from prior or current history of psychiatric, neurological or chronic somatic disorders participated in the second experiment. The participants were recruited from the Eötvös Lóránd University and were compensated with partial course credits. By applying the same criteria as for the other studies, we had to exclude five subjects: accuracy below 0.8 during the study phase (1), false alarm rate over 0.5 during the test phase (3) and overall uncorrected recognition rate below 0.05 (1). This left 30 participants (28 females, age: 21.1 ± 1.6). A sensitivity analysis indicated that the study could detect a minimum effect of *d* = 0.61 with 90% power and alpha set to 0.05. We obtained written informed consents from all participants, and the study was approved by the Hungarian Ethical Review Committee for Research in Psychology.

### Procedure

The general design, the amount of trials and the temporal characteristics of the experiment were the same as in Study 1. In Study 4 we applied textual cues, which directly indicated the category of the next picture (“old” vs. “new”). The sequence of trials during the study phase was fully randomized as in Study 3, thus the trial order was also unstructured in this experiment. Confidence ratings were collected during the test phase instead of remember/know responses.

### Statistical analyses

All procedures were the same as for Study 3.

### Results

During the study phase, overall accuracy of the participants was over 95% (see Table 1). The two‐way ANOVA showed no significant effects (STIMULUS CATEGORY: *F*[1,29] = 0.21, *p* > 0.5; EXPECTATION: *F*[1,29] = 0.03, *p* > 0.5; STIMULUS CATEGORY × EXPECTATION interaction: F[1,29] = 0.47, *p* > 0.1). The mean values of reaction time for the different categories showed the same pattern reported in the other experiments (see Fig. 3 for the mean reaction times in all studies). The two‐way ANOVA yielded a significant main effect for STIMULUS category (*F*[1,29] = 12.5, *p* = .001, η^2^ *G* = 0.019), and a significant main effect for expectation (*F*[1,29] = 4.31, *p* < 0.05, η^2^ *G* = 0.002), and a significant interaction too (expectation × STIMULUS category: *F*[1,29] = 5.59, *p* > 0.05, η^2^ *G* = 0.002). Post hoc *t*‐tests with the Tukey method revealed that the reaction times for expected familiar pictures were significantly shorter than the reaction times for any other category (*p* < 0.05), while the reaction times for other categories did not differ significantly (*p* > 0.1).

With respect to the test phase, comparison of confidence ratings for correctly recognized expected and unexpected pictures showed these ratings were not significantly different between unexpected and expected stimuli (*M* = 3.57, *SD* = 0.94 vs. *M* = 3.66, *SD* = 0.58; *t*(29) = 0. 90, *p* > 0.1, *d* = −0.11, CI [−0.42, 0.21]). The mean confidence ratings in Study 4 are visible in Fig. 6. The Shapiro–Wilk test showed a significant result (*p* < 0.001), thus we used a Wilcoxon signed rank test for the comparison, which showed no significant difference between stimulus categories (*p* = 0.7; Cliff's Delta = 0.02, CI [−0.27–0.31]). The Bayes factor of the *t*‐test comparing the mean confidence ratings for expected and unexpected novel pictures was 0.240. Thus, there was moderate support for the null hypothesis. The AUC for the ROC curve fitted to the responses to the expected picture category was 0.69 (95% CI: 0.55–0.83) and for the unexpected pictures was 0.73 (95% CI: 0.62–0.84). Thus, performance was over chance level (0.5). There was no significant difference between the AUC in the two conditions (*D* = −0.43, *p* = 0.67). The uncorrected hit rates are shown in Fig. 5. There was no significant difference between the corrected hit rates for unexpected and expected novel pictures either (*M* = 0.402, *SD* = 0.174 vs. *M* = 0.404, *SD* = 0.151; *t*(29) = 0.09, *p* > 0.5, *d* = 0.01, CI [−0.3, 0.26]). The Bayes factor of the *t*‐test comparing uncorrected recognition rates for expected and unexpected novel pictures was 0.195, showing moderate evidence in favor of the null hypothesis.

### Discussion

The results again support the idea that the task during the study phase was relatively easy and the participants paid attention to the cues. In this experiment, the confidence rates did not differ between expected and unexpected novel pictures. The overall uncorrected recognition rates did not differ between the categories, thus, none of the proposed hypotheses are supported by the outcome of this study (Table 2).

**Table 2:** Means and standard deviations for the variables of interest in the test phase in the behavioral study of Wittmann et al. (2007) and Studies 1–4

	Test phase									
	Uncorrected recognition rate (%)		Remember rate (%)			Know rate (%)			Mean confidence rating (1–5)	
	EN	UN	EN	UN	FA	EN	UN	FA	EN	UN
Wittmann *et al*. (2007)	58.6 ± 3.1	58.3 ± 3.1	21.1 ± 5	19.4 ± 4	4.2 ± 2	23.4 ± 2	26.6 ± 3	12.2 ± 2		
Study 1	35.7 ± 15.0	40.5 ± 13.2	7.37 ± 6.35	9.72 ± 9.16	7.2 ± 7.4	4.18 ± 4.25	6.99 ± 7.18	8.2 ± 6.3		
Study 2	37.6 ± 11.7	35.9 ± 13.0	11.84 ± 7.61	9.13 ± 7.00	4.5 ± 5.6	6.99 ± 5.77	7.47 ± 7.01	8.1 ± 6.6		
Study 3	35.3 ± 20.6	35.7 ± 17.4							3.22 ± 0.90	3.72 ± 0.40
Study 4	40.4 ± 15.1	40.2 ± 17.4							3.66 ± 0.58	3.57 ± 0.94

*Note*: EN = expected novel; FA = false alarm rate; UN = unexpected novel.

### Statistical analysis of all studies

Since the studies only differed in the type of cues and the trial structure during the study phase, we used a mixed ANOVA to judge if these manipulations had any effect on overall uncorrected recognition rates. For this model, we used EXPECTATION as a within‐subjects factor (expected/unexpected) and CUE TYPE (symbolic/verbal) and TRIAL STRUCTURE (yes/no) as between‐subject variables. A sensitivity analysis indicated that the study could detect a minimum effect of *f* = 0.17 with 90% power and alpha set to 0.05.

#### Results

There were not any significant main effects or significant interactions according to this model (CUE TYPE: *F*[1,114] = 0.41, *p* > 0.5, η^2^ *G* = 0.003; TRIAL STRUCTURE: *F*[1,114] = 0.03, *p* > 0.5, η^2^ *G* < 0.001; EXPECTATION: *F*[1,114] = 0.72, *p* > 0.2, η^2^ *G* < 0.001; CUE TYPE × EXPECTATION: *F*[1,114] = 3.33, *p* = 0.07, η^2^ *G* = 0.003; CUE TYPE × TRIAL STRUCTURE: *F*[1,114] = 1.29, *p* > 0.2, η^2^ *G* = 0.01; EXPECTATION × TRIAL STRUCTURE: *F*[1,114] = 0.57, *p* > 0.4, η^2^ *G* < 0.001; CUE TYPE × TRIAL STRUCTURE × EXPECTATION: *F*[1,114] = 2.36, *p* > 0.1, η^2^ *G* = 0.002).

## GENERAL DISCUSSION

Our main goal was to replicate the behavioral results of Wittmann *et al*. (2007), specifically that the anticipation of novel stimuli enhances recognition performance for these stimuli. However, we did not see this effect in any of our four studies. A classic theory, the novelty/encoding hypothesis suggests that novel stimuli are recognized with a higher probability (Tulving *et al*., 1996). Even though this conclusion is not unequivocally supported by studies with human participants (Barto *et al*., 2013; Dobbins *et al*., 1998; Reichardt *et al*., 2020), animal studies produced persuasive results (Lisman & Grace, 2005). Based on this literature, the idea was articulated that novelty – similarly to rewards – causes dopamine release from midbrain neurons and enhances memory formation in the hippocampus (Düzel *et al*., 2010; Lisman, Grace & Duzel, 2011). Since it was known that the expectation of rewards also elicits the activation of midbrain dopaminergic neurons, it was suggested that the same holds for novelty (Düzel *et al*., 2010; Wittmann *et al*., 2007). We propose that even if this hypothesis may be true, the paradigm used in our studies is not suitable to thoroughly examine this question. Novelty in this study is behaviorally irrelevant; therefore, a nervous system shaped by evolution to efficiently guide behavior should not prioritize these stimuli (Clark, 2013; Friston, 2010). Our data is in line with this conclusion, as we did not find a single outcome showing better recognition memory for expected novel pictures.

On the first sight, this seems to further complicate the literature on the memory effects of novelty, yet predictive coding theories may help organize the findings into an explanatory framework. Predictive coding theories of brain function suggest that the brain is constantly trying to predict its inputs and when these predictions differ from the observed outcomes, prediction errors are generated in order to update internal models of the environment and ultimately increase the accuracy of subsequent predictions (Clark, 2013; Friston, 2005, 2010). Predictive coding has already been suggested to play a key role in memory processes, and prediction error signals following unexpected events are assumed to facilitate learning (Henson & Gagnepain, 2010; Reichardt *et al*., 2020; Van Kesteren, Ruiter, Fernández & Henson, 2012). Nevertheless, novelty and unexpectedness are usually not adequately distinguished at the conceptual level, which may lead to conflicting findings (Barto *et al*., 2013; Reichardt *et al*., 2020). Human studies that examine the memory effects of novelty often apply meaningless novel stimuli such as algorithmically generated fractals (Schomaker & Meeter, 2012, 2018) or objects that are irrelevant with respect to the memory task (Schott *et al*., 2004; Wittmann *et al*., 2007). Therefore, in human studies, novel stimuli might become less relevant and more expected during the experiment, while in animal studies, novelty and high unexpectedness always coincide. In the human and the animal literature, manipulations of novelty and expectation are confounded, and in fact, the latter but not the former might be the key factor mediating learning (Reichardt *et al*., 2020).

Predictive coding theories suggest that expectations shape memory formation and unexpected stimuli are more likely to be recognized later (Henson & Gagnepain, 2010; Van Kesteren *et al*., 2012). In this experiment, the cues during the study phase may generate expectations as to what type of stimulus will appear next. These expectations are sometimes contradicted when familiar or novel pictures appear unexpectedly. If predictive coding is a general strategy for information processing in the nervous system, its principles should be demonstrable in relation to memory processes (Reichardt *et al*., 2020; Schomaker & Meeter, 2015; Van Kesteren *et al*., 2012). In this paradigm, we expected to see higher recognition rates for unexpected novel pictures, or that remember/know responses and confidence ratings show that unexpected novel pictures are more likely to be remembered or recognized with a higher confidence rating. This result only appears in one of our studies, which lead us to conclude that the paradigm is not suited to consistently reveal a difference in recognition memory for these stimulus categories. A reason for this may be that there is only one source of unexpectedness in the study and if we consider it as a continuous variable, we can assume that it gradually decreases during the experiment. The umpteenth unexpected picture may not be as unexpected as the first one. We propose that the higher overall recognition rate for unexpected pictures in Study 1 lends some support to the idea that human memory operates on the basis of predictive coding, however, the usage of the paradigm to further elucidate these regularities is not advisable. Yet using similar tasks, other researchers have been able to consistently show that unexpected novel stimuli enhance recollection, while expected novelty enhances familiarity on a recognition memory test delayed by less than an hour (Kafkas, 2021; Kafkas & Montaldi, 2015, 2018). We suspect that this effect is erased by post‐encoding memory processes that take place within a 24 h delay used in our experiments.

The analysis of reaction times was used as a proxy to confirm the effect of the cues. Although these analyses are also somewhat inconsistent, we believe that this is due to the small magnitude of these effects. During pilot studies, participants remarked that since the cues were not always correct, and the visual discrimination task was relatively easy, they simply ignored the cues and paid attention only to the pictures. Even though these reports do not exclude the possibility that behaviorally relevant expectations were elicited by these cues, we tried two methods to encourage the consideration of the cues. We fixed the first 10% of the trials in Study 1 and 2 so that the cues were always correct at the start of the task (24 trials). We used verbal cues in study 2 and 4 in the hope that the inherent association between the meaning of the words and the predicted category will make the participants pay heed to the cues. Interestingly, only the study using symbolic cues and structured trials produced a significant difference between the correct recognition for expected and unexpected novel pictures. It is tempting to speculate that the need to learn the association between the cues and the picture categories boosted the memory enhancing effect of unexpectedness to a detectable level, however, the mixed model ANOVA we used to analyze the effect of the quality of the cue and the structuring of trials throughout the four studies showed no significant effect of either one. Another speculative hypothesis about the effect of the cues is that the contingencies which prove to be trustworthy may be enduring and may influence memory formation longer than unreliable associations. In other words, when the expectations prove useful, unexpected events become more memorable. Even though this hypothesis is not directly supported by the results of these studies, it may be worthwhile to further explore this possibility. However, it is also worth noting, that in animal studies, novelty manipulations usually work in greater timeframes and there are also studies done with humans where similar novelty manipulations (i.e., exploration of a virtual environment) produced discernible effects on learning (Schomaker, van Bronkhorst & Meeter, 2014). Thus, another explanation for our null results and the general inconsistency of studies on the memory effects of novelty is that novelty simply does not work on the timeframe set by experimental psychology.

In sum, our experiments failed to replicate the main behavioral result of the original study by Wittmann and colleagues (2007). We suspect that this is because the pictures in this study are landscapes, often without any prominent feature and they are also irrelevant during the task of the study phase. This results in low recognition rates and the differences that should appear are thus masked. Another possible explanation is that in the original study participants anticipated memory testing on the next day appointment. In the original paper, there is no mention of active misdirection of the participants, while we found during piloting that participants suspected memory testing after the first session. In our view, it is central to the paradigm that learning is incidental during the study phase, else the effects in recognition memory will not be attributable to the anticipation of novelty, but instead will be the anticipation of an important stimulus that should be remembered later. A recent study showed that longer viewing time increased memory performance to a greater degree in an intentional learning condition than in an incidental condition (Helbing, Draschkow & Võ, 2020). Thus, anticipation of a stimulus to learn in an intentional learning paradigm could possibly improve performance. This could also explain the difference visible in corrected hit rates between the original study and the four studies reported in this paper: intentional learning enhances recognition performance for visual stimuli (Noldy, Stelmack & Campbell, 1990). This would mean that the memory effect attributed to the anticipation of novelty is in fact the effect of intentional attention allocation. Another factor in the nonreplication may be the pacing of stimulus presentation. In the original study, intertrial intervals in the study phase were optimized for the collection of imaging data and this timing was used in the behavioral study. We markedly decreased this interval (1,500–4,500 to 1,000–2,000 ms) in order to reduce the overall length of the study. This however resulted in a more rapid stimulus presentation than in the original study, which may influence encoding processes and ultimately, recognition memory. Since we did not use physiological measures during our studies we are reluctant to speculate on the imaging findings of the original study. Anticipation of novelty may activate dopaminergic areas, yet not necessarily lead to demonstrable effects in recognition memory. The link between novelty processing and the dopaminergic midbrain should be further explored by future studies.

Some differences in memory performance between the expected and unexpected categories still surfaced sporadically in our studies, although these were in favor of the unexpected category, lending some support to the idea that the principles of predictive coding also affect memory processes. Unexpected events generate prediction errors in the appropriate systems, and these drive the updating of the inner model, which results in a demonstrable recognition memory enhancement for unexpected items in this paradigm. Overall, the series of studies we conducted point to the direction that expectation of novelty does not have a consistent effect on subsequent recognition memory performance (at least in the paradigm we used); thus, new paradigms and maybe even new directions are needed to reveal how novelty and expectation impact memory formation. We hope that our results pique the curiosity of other researchers in the field and encourage them to expand the horizon of novelty manipulations in memory research. We believe that working out the memory effects of novelty and expectation is one of the most interesting undertakings in the broader field of memory research, not least because it holds the enticing promise of development in educational methods.

The project was supported by the Hungarian Scientific Research Fund (NKFI/OTKA FK 128100 and NKFIH‐1157‐8/2019‐DT) of the National Research, Development and Innovation Office. BP was supported by the BME‐Biotechnology FIKP grant of EMMI (BME FIKP‐BIO), and by the National Research, Development and Innovation Office (NKFI/OTKA K 128599). We would like to thank Anna Kincses, Roland Tóth, Janka Miklós‐Kovács and Dániel Fehér for their suggestions and their help with data collection.

## References

[sjop12807-bib-0001] Adcock, R.A. , Thangavel, A. , Whitfield‐Gabrieli, S. , Knutson, B. & Gabrieli, J.D. (2006). Reward‐motivated learning: Mesolimbic activation precedes memory formation. Neuron, 50, 507–517. 16675403 10.1016/j.neuron.2006.03.036

[sjop12807-bib-0002] Andraszewicz, S. , Scheibehenne, B. , Rieskamp, J. , Grasman, R. , Verhagen, J. & Wagenmakers, E. J. (2015). An introduction to Bayesian hypothesis testing for management research. Journal of Management, 41, 521–543.

[sjop12807-bib-0003] Barto, A. , Mirolli, M. & Baldassarre, G. (2013). Novelty or surprise? Frontiers in Psychology, 4, 1–15. 24376428 10.3389/fpsyg.2013.00907 PMC3858647

[sjop12807-bib-0004] Clark, A. (2013). Whatever next? Predictive brains, situated agents, and the future of cognitive science. Behavioral and Brain Sciences, 36, 181–204. 23663408 10.1017/S0140525X12000477

[sjop12807-bib-0005] Davis, C.D. , Jones, F.L. & Derrick, B.E. (2004). Novel environments enhance the induction and maintenance of long‐term potentiation in the dentate gyrus. Journal of Neuroscience, 24, 6497–6506. 15269260 10.1523/JNEUROSCI.4970-03.2004 PMC6729872

[sjop12807-bib-0006] Dobbins, I.G. , Kroll, N.E.A. , Yonelinas, A.P. & Liu, Q. (1998). Distinctiveness in recognition and free recall: The role of recollection in the rejection of the familiar. Journal of Memory and Language, 38, 381–400.

[sjop12807-bib-0007] Düzel, E. , Bunzeck, N. , Guitart‐Masip, M. & Düzel, S. (2010). NOvelty‐related motivation of anticipation and exploration by dopamine (NOMAD): Implications for healthy aging. Neuroscience and Biobehavioral Reviews, 34, 660–669. 19715723 10.1016/j.neubiorev.2009.08.006

[sjop12807-bib-0008] Düzel, E. , Habib, R. , Rotte, M. , Guderian, S. , Tulving, E. & Heinze, H.‐J. (2003). Human hippocampal and parahippocampal activity during visual associative recognition memory for spatial and nonspatial stimulus configurations. The Journal of Neuroscience: The Official Journal of the Society for Neuroscience, 23, 9439–9444. 14561873 10.1523/JNEUROSCI.23-28-09439.2003 PMC6740564

[sjop12807-bib-0010] Erdfelder, E. , Faul, F. , Buchner, A. & Lang, A.G. (2009). Statistical power analyses using G*power 3.1: Tests for correlation and regression analyses. Behavior Research Methods, 41, 1149–1160. 19897823 10.3758/BRM.41.4.1149

[sjop12807-bib-0011] Frank, D. & Kafkas, A. (2021). Expectation‐driven novelty effects in episodic memory. Neurobiology of Learning and Memory, 183, 107466. 34048914 10.1016/j.nlm.2021.107466

[sjop12807-bib-0012] Friston, K. (2005). A theory of cortical responses. Philosophical Transactions of the Royal Society B: Biological Sciences, 360, 815–836. 10.1098/rstb.2005.1622 PMC1569488 15937014

[sjop12807-bib-0013] Friston, K. (2010). The free‐energy principle: A unified brain theory? Nature Reviews Neuroscience, 11, 127–138. 20068583 10.1038/nrn2787

[sjop12807-bib-0014] Helbing, J. , Draschkow, D. & Võ, M.L.H. (2020). Search superiority: Goal‐directed attentional allocation creates more reliable incidental identity and location memory than explicit encoding in naturalistic virtual environments. Cognition, 196, 104147. 32004760 10.1016/j.cognition.2019.104147

[sjop12807-bib-0015] Henson, R.N. & Gagnepain, P. (2010). Predictive, interactive multiple memory systems. Hippocampus, 20, 1315–1326. 20928831 10.1002/hipo.20857

[sjop12807-bib-0016] Kafkas, A. (2021). Encoding‐linked pupil response is modulated by expected and unexpected novelty: Implications for memory formation and neurotransmission. Neurobiology of Learning and Memory, 180, 107412. 33609740 10.1016/j.nlm.2021.107412

[sjop12807-bib-0017] Kafkas, A. & Montaldi, D. (2015). Striatal and midbrain connectivity with the hippocampus selectively boosts memory for contextual novelty. Hippocampus, 25, 1262–1273. 25708843 10.1002/hipo.22434 PMC4672698

[sjop12807-bib-0018] Kafkas, A. & Montaldi, D. (2018). Expectation affects learning and modulates memory experience at retrieval. Cognition, 180, 123–134. 30053569 10.1016/j.cognition.2018.07.010 PMC6191926

[sjop12807-bib-0020] Köhler, S. , Danckert, S. , Gati, J.S. & Menon, R.S. (2005). Novelty responses to relational and non‐relational information in the hippocampus and the parahippocampal region: A comparison based on event‐related fMRI. Hippocampus, 15, 763–774. 15999342 10.1002/hipo.20098

[sjop12807-bib-0021] Kormi‐Nouri, R. , Nilsson, L.G. & Ohta, N. (2005). The novelty effect: Support for the novelty‐encoding hypothesis. Scandinavian Journal of Psychology, 46, 133–143. 15762941 10.1111/j.1467-9450.2005.00443.x

[sjop12807-bib-0022] Kumaran, D. & Maguire, E.A. (2007). Match mismatch processes underlie human hippocampal responses to associative novelty. Journal of Neuroscience, 27, 8517–8524. 17687029 10.1523/JNEUROSCI.1677-07.2007 PMC2572808

[sjop12807-bib-0023] Kumaran, D. & Maguire, E.A. (2006). An unexpected sequence of events: Mismatch detection in the human hippocampus. PLoS Biology, 4, 2372–2382. 10.1371/journal.pbio.0040424 PMC1661685 17132050

[sjop12807-bib-0025] Li, S. , Cullen, W.K. , Anwyl, R. & Rowan, M.J. (2003). Dopamine‐dependent facilitation of LTP induction in hippocampal CA1 by exposure to spatial novelty. Nature Neuroscience, 6, 526–531. 12704392 10.1038/nn1049

[sjop12807-bib-0026] Lisman, J. , Grace, A.A. & Duzel, E. (2011). A neoHebbian framework for episodic memory; role of dopamine‐dependent late LTP. Trends in Neurosciences, 34, 536–547. 21851992 10.1016/j.tins.2011.07.006 PMC3183413

[sjop12807-bib-0027] Lisman, J.E. & Grace, A.A. (2005). The hippocampal‐VTA loop: Controlling the entry of information into long‐term memory. Neuron, 46, 703–713. 15924857 10.1016/j.neuron.2005.05.002

[sjop12807-bib-0028] Mathot, S. , Schreij, D. & Theeuwes, J. (2012). OpenSesame: An open‐source, graphical experiment builder for the social sciences. Behavior Research Methods, 44, 314–324. 22083660 10.3758/s13428-011-0168-7 PMC3356517

[sjop12807-bib-0029] Morey, R. D. , Rouder, J. N. , Jamil, T. & Morey, M. R. D. (2015). Package ‘bayesfactor’. Retrieved 10 June 2015 from http://cran/r‐projectorg/web/packages/BayesFactor/BayesFactor

[sjop12807-bib-0030] Noldy, N.E. , Stelmack, R.M. & Campbell, K.B. (1990). Event‐related potentials and recognition memory for pictures and words: The effects of intentional and incidental learning. Psychophysiology, 27, 417–428. 2236443 10.1111/j.1469-8986.1990.tb02337.x

[sjop12807-bib-0031] Poppenk, J. , Köhler, S. & Moscovitch, M. (2010). Revisiting the novelty effect: When familiarity, not novelty, enhances memory. Journal of Experimental Psychology: Learning Memory and Cognition, 36, 1321–1330. 20804299 10.1037/a0019900

[sjop12807-bib-0032] Quent, J.A. , Henson, R.N. & Greve, A. (2021). A predictive account of how novelty influences declarative memory. Neurobiology of Learning and Memory, 179, 107382. 10.1016/j.nlm.2021.107382 33476747 PMC8024513

[sjop12807-bib-0033] R Core Team . (2020). R: A language and environment for statistical computing. R Foundation for Statistical Computing, Vienna, Austria. Retrieved 27 October 2021 from https://www.R‐project.org/

[sjop12807-bib-0034] Ranganath, C. & Rainer, G. (2003). Cognitive neuroscience: Neural mechanisms for detecting and remembering novel events. Nature Reviews Neuroscience, 4, 193–202. 12612632 10.1038/nrn1052

[sjop12807-bib-0035] Reichardt, R. , Polner, B. & Simor, P. (2020). Novelty manipulations, memory performance, and predictive coding: The role of unexpectedness. Frontiers in Human Neuroscience, 14, 1–11. 32410975 10.3389/fnhum.2020.00152 PMC7201021

[sjop12807-bib-0036] Robin, X. , Turck, N. , Hainard, A. , Tiberti, N. , Lisacek, F. , Sanchez, J.C. *et al*. (2011). pROC: An open‐source package for R and S+ to analyze and compare ROC curves. BMC Bioinformatics, 12, 1–8. 10.1186/1471-2105-12-77 21414208 PMC3068975

[sjop12807-bib-0037] RStudio Team . (2015). RStudio: Integrated development for R. RStudio, Inc.: Boston, MA. Retrieved 27 October 2021 from http://www.rstudio.com/

[sjop12807-bib-0038] Schomaker, J. & Meeter, M. (2012). Novelty enhances visual perception. PLoS One, 7, e50599. 10.1371/journal.pone.0050599 23227190 PMC3515594

[sjop12807-bib-0039] Schomaker, J. & Meeter, M. (2015). Short‐ and long‐lasting consequences of novelty, deviance and surprise on brain and cognition. Neuroscience and Biobehavioral Reviews, 55, 268–279. 25976634 10.1016/j.neubiorev.2015.05.002

[sjop12807-bib-0040] Schomaker, J. & Meeter, M. (2018). Predicting the unknown: Novelty processing depends on expectations. Brain Research, 1694, 140–148. 10.1016/j.brainres.2018.05.008 29758180

[sjop12807-bib-0041] Schomaker, J. , van Bronkhorst, M.L.V. & Meeter, M. (2014). Exploring a novel envi‐ronment improves motivation and promotes recall of words. Frontiers in Psychology, 5, 918. 25191297 10.3389/fpsyg.2014.00918 PMC4138787

[sjop12807-bib-0042] Schott, B.H. (2006). The dopaminergic midbrain participates in human episodic memory formation: Evidence from genetic imaging. Journal of Neuroscience, 26, 1407–1417. 16452664 10.1523/JNEUROSCI.3463-05.2006 PMC6675495

[sjop12807-bib-0043] Schott, B. H. , Sellner, D. B. , Lauer, C.‐J. , Habib, R. , Frey, J. U. , Guderian, S. *et al*. (2004). Activation of midbrain structures by associative novelty and the formation of explicit memory in humans. Learning & Memory, 11, 383–387. 15254215 10.1101/lm.75004

[sjop12807-bib-0044] Straube, T. , Korz, V. , Balschun, D. & Uta Frey, J. (2003). Requirement of β‐adrenergic receptor activation and protein synthesis for LTP‐reinforcement by novelty in rat dentate gyrus. The Journal of Physiology, 552, 953–960. 12937286 10.1113/jphysiol.2003.049452 PMC2343450

[sjop12807-bib-0045] Straube, T. , Korz, V. & Frey, J.U. (2003). Bidirectional modulation of long‐term potentiation by novelty‐exploration in rat dentate gyrus. Neuroscience Letters, 344, 5–8. 12781908 10.1016/s0304-3940(03)00349-5

[sjop12807-bib-0046] Tulving, E. & Kroll, N. (1995). Novelty assessment in the brain and long‐term memory encoding. Psychonomic Bulletin & Review, 2, 387–390. 24203720 10.3758/BF03210977

[sjop12807-bib-0047] Tulving, E. , Markowitsch, H.J. , Craik, F.I.M. , Habib, R. & Houle, S. (1996). Novelty and familiarity activations in PET studies of memory encoding and retrieval. Cerebral Cortex, 6, 71–79. 8670640 10.1093/cercor/6.1.71

[sjop12807-bib-0048] Van Kesteren, M.T.R. , Ruiter, D.J. , Fernández, G. & Henson, R.N. (2012). How schema and novelty augment memory formation. Trends in Neurosciences, 35, 211–219. 22398180 10.1016/j.tins.2012.02.001

[sjop12807-bib-0050] Wickham, H. (2016). ggplot2: Elegant graphics for data analysis. New York: Springer‐Verlag.

[sjop12807-bib-0051] Wittmann, B.C. , Bunzeck, N. , Dolan, R.J. & Düzel, E. (2007). Anticipation of novelty recruits reward system and hippocampus while promoting recollection. NeuroImage, 38, 194–202. 17764976 10.1016/j.neuroimage.2007.06.038 PMC2706325

[sjop12807-bib-0053] Yonelinas, A.P. , Aly, M. , Wang, W.C. & Koen, J.D. (2010) Recollection and familiarity: Examining controversial assumptions and new directions. Hippocampus, 20, 1178–1194. 20848606 10.1002/hipo.20864 PMC4251874

[sjop12807-bib-0052] Yonelinas, A.P. , Dobbins, I. , Szymanski, M.D. , Dhaliwal, H.S. & King, L. (1996) Signal‐detection, threshold, and dual‐process models of recognition memory: ROCs and conscious recollection. Consciousness and Cognition, 5, 418–441. 9063609 10.1006/ccog.1996.0026

